# Diagnosis of approximal caries in children with convolutional neural networks based detection algorithms on radiographs: A pilot study

**DOI:** 10.2340/aos.v84.42599

**Published:** 2025-01-06

**Authors:** Zeynep Seyda Yavsan, Hediye Orhan, Enes Efe, Emrehan Yavsan

**Affiliations:** a Department of Pediatric Dentistry, Tekirdag Namik Kemal University, Tekirdag, Turkey; b Department of Computer Engineering, Necmettin Erbakan University, Konya, Turkey; c Department of Electrical and Electronics Engineering, Hitit University, Corum, Turkey; d Electronic and Department of Electronics and Automation, Tekirdag Namik Kemal University, Tekirdag, Turkey

**Keywords:** Artificial intelligence, dental caries, machine learning, periapical radiography, pediatric dentistry

## Abstract

**Objectives:**

Approximal caries diagnosis in children is difficult, and artificial intelligence-based research in pediatric dentistry is scarce. To create a convolutional neural network (CNN)-based diagnostic system for the prompt and efficient identification of approximal caries in pediatric patients aged 5–12 years.

**Materials and methods:**

Pediatric patients’ digital periapical radiographic images were collected to create a unique dataset. Various augmentation methods were used, and approximal caries in the augmented images were labeled by a pediatric dentist to minimize labeling errors. The dataset consisted of 830 data labeled for approximal caries on 415 images, which were divided into 80% training and 20% testing sets. After comparing 13 detection algorithms, including the latest YOLOv8, the most appropriate one was selected for the proposed system, which was then evaluated based on various performance metrics.

**Results:**

The proposed detection system achieved a precision of 91.2%, an accuracy of 90.8%, a recall of 89.3%, and an F1 value of 90.24% after 300 iterations, utilizing a learning rate of 0.01.

**Conclusion:**

Approximal caries has been successfully detected with the developed system. Future efforts will focus on augmenting the dataset and expanding the sample size to enhance the efficacy of the system.

## Introduction

Dental caries is a common chronic infectious oral disease affecting children, teenagers, and adults worldwide [1]. Early diagnosis and rapid treatment of dental caries are extremely important, especially in the primary dentition period. The health of primary teeth is important as it is the foundation of permanent teeth [2]. The enamel layer of primary teeth is thinner than permanent teeth and dental plaque accumulates more easily [3]. These features of primary teeth make them more vulnerable to caries and lead to the progression of caries. Untreated caries; can reach enamel, dentin, and even pulp tissue. As a result, severe pain may occur, and this may lead to the premature loss of the primary tooth [4]. It is also well known that untreated dental caries adversely affect the oral health-related quality of life of children [5].

Visual and radiographic examination are traditional methods used in the detection of dental caries [2]. Although these methods are considered reliable, the diagnostic accuracy of visual and radiographic examination is significantly affected by the various anatomical morphologies of the teeth and the experience of the dentist [6]. Especially in primary molars, it can be difficult to detect and evaluate the depth of caries by visual inspection due to the wide proximal contacts [7]. Although dental radiography, which includes panoramic, periapical, and bitewing views, as well as dental probing, is widely recognized as reliable diagnostic tools for detecting dental caries, the screening and final diagnosis of this condition often relies on empirical evidence. This challenge can be facilitated by developing artificial intelligence (AI) technologies. Dental caries in radiological images can be detected quickly and effectively with convolutional neural networks (CNN). Thus, the diagnostic accuracy can be increased. Unlike simple and traditional methods, deep CNN algorithms can perform edge detection very efficiently with their hierarchical features [8, 9].

Although the use of artificial neural networks in conservative dentistry has increased, it is still not very common [6]. Neural networks are mathematical models that mimic the functioning of the brain. They are computing systems consisting of several interconnected processing elements. They process the information given by external inputs and generate a dynamic status response [10, 11]. Neural networks are already used in various medical fields, including medical diagnostics. However, the use of neural networks for caries detection in dentistry is relatively limited [10, 12, 13]. In fact, within the scope of the literature research in this study, according to the knowledge of the authors, there was no study in which approximal caries was detected in primary teeth using periapical radiographs. Thus, in this study, we evaluated the success of deep-learning-based CNN methods with various detection algorithms in the approximal caries detection of the primary teeth of pediatric patients as depicted on periapical radiographs.

## Materials and methods

### Image preprocessing and augmentation

**Figure 1:**
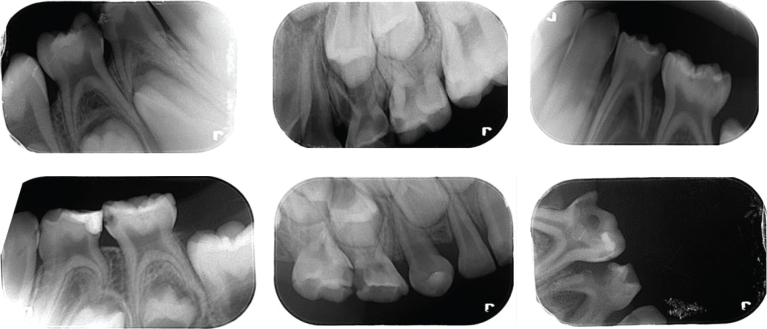
Some of the collected radiographic images.

This work was approved by Tekirdag Namik Kemal University Ethics Committee (approval No. 2022.240.12.18) and carried out at the Department of Pediatric Dentistry, Faculty of Dentistry Tekirdağ Namık Kemal University, Turkey. To detect approximal caries in children, a unique dataset was created by collecting periapical radiographic images. All radiographs with aproximal lesions, excluding unclear ones, were included in the study. Only non-clear radiographs were excluded from the dataset. Hence, a unique dataset of periapical radiographs was obtained. Some of these radiographic images are given in Figure 1.

**Figure 2:**
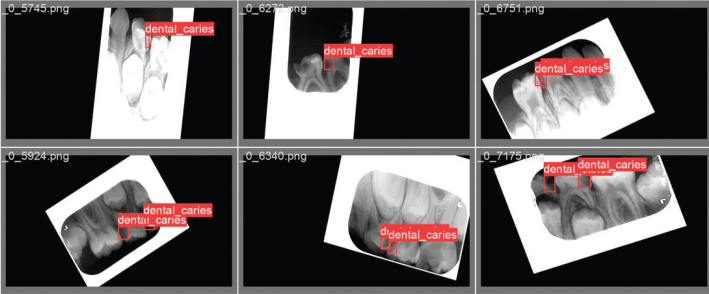
Radiographic image samples labeled by a pediatric dentist.

The data obtained from the various radiographic images in the dataset were augmented using the Python programming language and the Keras library. Data augmentation process: the images were realized by rotating the x‑y axes and changing the color tones. With the implementation of the data augmentation process, a total of 415 radiographic images were obtained. The augmented data are labeled in .txt format. In the labeling process, the approximal caries in the radiographic images are framed with a red box, and the coordinates of the caries are indicated. Two pediatric dentists served as observers during this process. The reliability of the observers was evaluated comparatively. After 10 days from the first observer’s assessment, the same observer performed re-labeling, and 96.6% consistency was observed between this labeling and the initial one. The second observer then labeled and 98.3% consistency was observed between the first observer. Figure 2 shows a few of the radiographic images labeled by pediatric dentists who specialize in pediatrics at the university.

Consistent with established practices [14, 15], the dataset was partitioned into 80% training data and 20% test data. The training data were allocated for the development of the caries detection model, while the test data were reserved for evaluating the trained model’s performance.

### Proposed detection algorithm and its architecture

**Figure 3:**
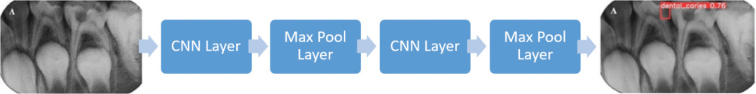
General YOLO architecture [24].

Transfer learning was used to detect dental caries. The You Only Look Once (YOLO) algorithm offers higher accuracy than other detection algorithms in the literature [16]. Hence, it was decided to train with the YOLO detection algorithm for the presented study. In YOLO algorithms, object detection is realized via CNN [14, 17]. The general network structure of the YOLO algorithm is illustrated in Figure 3 [14, 15].

YOLO is an object-tracking algorithm that processes images by dividing them into regions. YOLO operates faster than other object-tracking algorithms because it passes the image through neural networks in a single instance. The slowness of other algorithms is due to their method of identifying potential object areas and passing each one through neural networks separately. As the number of operations on the image increases, the speed of the algorithms decreases. During the object detection phase, YOLO utilizes bounding boxes. The center point of the object within the bounding box is located. The size, coordinates, and confidence score of the object within the bounding box can be accessed. These values for size, coordinates, and confidence score are held as prediction vectors. Separate prediction vectors are created for each bounding box.

In this study, the Feature Pyramid Network (FPN) was utilized for YOLOv3. For YOLOv4, instead of FPN, Spatial Attention Module (SAM), Path Aggregation Network (PAN), and Spatial Pyramid Pooling (SPP) were employed. In SAM, maximum pooling and average pooling operations are applied separately, and the results are fed into a sigmoid activation function. YOLOv5 is a PyTorch implementation, utilizing a CSP backbone and PANet neck. The head part of YOLOv5 is the same as YOLOv4. In the intermediate layers of YOLOv5, leaky ReLU is used as the activation function, while the final layer employs a sigmoid function. Stochastic Gradient Descent (SGD) is preferred as the optimizer. YOLOv7 uses the YOLOv4 architecture; thus, it includes SPP, which allows for better predictions of objects of varying sizes. Additionally, YOLOv7 involves less computation and fewer parameters. YOLOv6 utilizes the PyTorch framework, and YOLOv8 is built on the CSP Darknet architecture.

The CNN architecture underlying the YOLO algorithm takes an image as input. The input passes through convolutional, pooling, and fully connected layers (FC) for training. Here, the convolutional layer is the first layer that deals with the image. An image consists of matrices of pixels carrying specific values. In this layer, a filter smaller than the original image size moves over the image, attempting to capture certain features. The matrix created as a result of the filter application is called a feature map. The parameters learned in the CNN are the values in these matrices. The model continuously updates these values and begins to learn the features better. The purpose of the pooling layer is to reduce the size of the input. This reduces computational intensity and focuses on important features by disregarding unnecessary ones. There are no learned parameters in this layer of the network. The FC layer is usually found at the end of the CNN architecture. This layer is used to optimize targets such as class scores. In this layer, the matrix resulting from convolutional and pooling processes is flattened into a vector.

There are various variants of this network structure. In the presented study, training was carried out on 13 variants of the YOLO algorithm. To determine the algorithm that gives the highest accuracy for the original dataset in this study, training was made on the proposed YOLO versions. The YOLO versions being trained are YOLOv4, YOLOv5x, YOLOv5s, YOLOv5m, YOLOv5l, YOLOv6s, YOLOv7x, YOLOv8n, YOLOv8s, YOLOv8m, and YOLOv8x. The trainings were carried out in 300 iterations and four batches with an image size of 640 × 480. To ensure consistency and fair comparison across all YOLO variants, the same 80% training and 20% testing split of our 415-image dataset was utilized for each algorithm. This approach allowed us to evaluate the performance of all 13 YOLO variants under identical conditions, thereby providing a robust basis for selecting the most appropriate algorithm for our proposed system. The accuracies of the proposed YOLO detection algorithms are given in Table 1. For the YOLO models, the accuracy value is symbolized by the mAP parameter. The mAP parameter is calculated on the mean precision (AP) for each class. This parameter is formulated with (1). In equation (1), the number of classes is symbolized by n, and the mean precision of the kth class is symbolized by APk.


$$\mathrm{mAP}\;=\;\frac{1}{n}•\begin{array}{c} k=n\\ k=1 \end{array}AP_{k}$$ (1)

**Table 1:** The comparison of proposed detection algorithms over various metrics. The YoloV5-s with a mAP of 90.8 demonstrates the highest performance, surpassing other Yolo versions. Its high recall (0.893) and precision (0.912) emphasize its effectiveness.

Proposed algorithm	Mean average precision (mAP) (%)	Epoch	Batch	Recall	Precision
YoloV4	83.25	300	4	0.86	0.80
YoloV5-x	86.7	300	4	0.871	0.863
YoloV5-m	88.5	300	4	0.843	0.909
YoloV5-m	86	300	1	0.857	0.882
YoloV5-l	83.9	300	4	0.857	0.847
YoloV5-s	87.6	300	4	0.832	0.886
**YoloV5-s**	**90.8**	**300**	**1**	**0.893**	**0.912**
YoloV6-s	86.17	300	4	0.852	0.875
YoloV7-x	86.9	300	4	0.85	0.862
YoloV8-s	85.5	300	4	0.80	0.92
YoloV8-x	88.7	300	4	0.857	0.88
YoloV8-m	85.9	300	4	0.807	0.858
YoloV8-n	86.2	300	4	0.842	0.849

**Figure 4:**
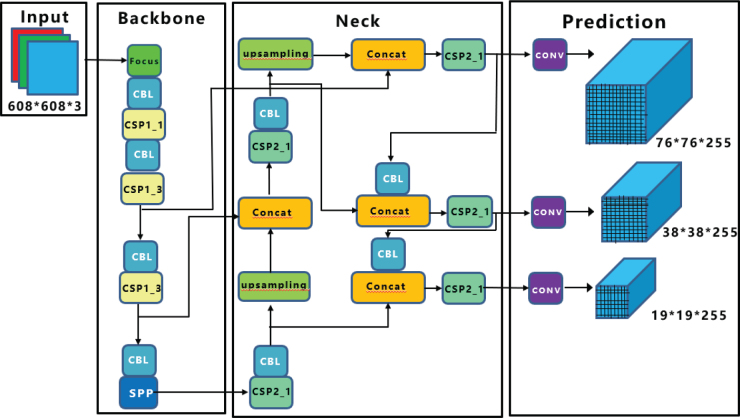
YOLOv5 architecture consisting of an input layer with mosaic augmentation combining four different X-ray images, a backbone layer with CSPDarknet53, a neck layer using PANet with upsampling and concatenation, and a prediction layer with outputs at three scales [25].

The highest accuracy with 90.8% in Table 1 was achieved with the YOLOv5s algorithm. Thus, the YOLOv5s algorithm was adopted in this study. The block diagram of the YOLOv5s algorithm is illustrated in Figure 4.

In the input layer of YOLOv5, the mosaic data augmentation method randomly selects and combines four different X-ray images from the dataset into a single training image, creating a diverse learning environment that enriches the background information and improves small object detection capabilities. The backbone layer consists of the focus module and the CSPDarknet53 structure. This layer is mainly used to extract essential features from the input image. The neck layer is responsible for the transmission of these features and speeds up feature fusion. It assists in describing the same object in various sizes and scales. In the prediction layer, objects of various sizes are detected [18].

The small batch values in Table 1 provide better training with less data in each epoch. Therefore, retraining was carried out on the models with high accuracy with 300 epochs and 1 batch values to increase the accuracy. 90.8% accuracy was achieved with the YOLOv5s algorithm in this retraining process.

## Results

### Training performance

**Figure 5:**
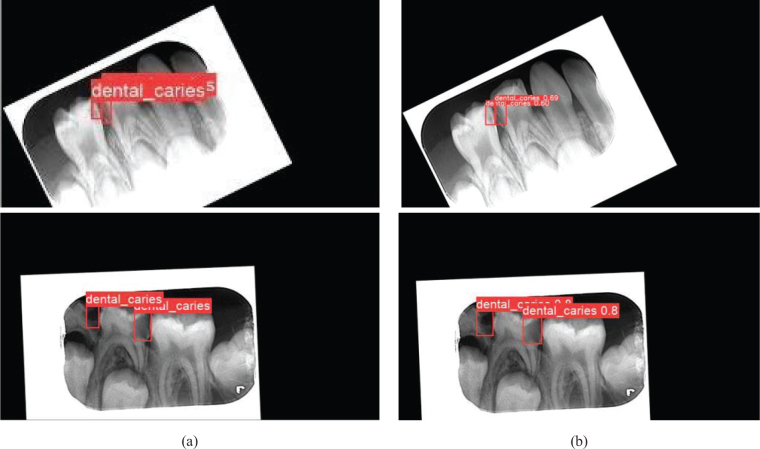
Evaluation of the detection performance of the proposed AI-based model with a comparison between labeling and testing stages on the same radiographic images. (a) Radiographic images labeled by the pediatric dentist before training and testing process and (b) the output images from the test.

The detection model was tested with the test dataset over the best weight values obtained as a result of 300 iterations of training. In Figure 5, the left-side images (Figure 5a) are the labeled test data, and the right-side images (Figure 5b) are the detection images predicted by the trained model.

**Figure 6:**
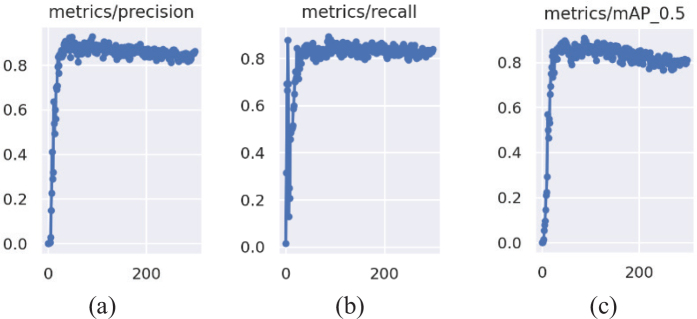
Variations of the performance criteria depending on the iterations count. (a) P, (b) R, and (c) mAP metrics variation over 300 iterations.

To evaluate the performance of the detection model, we calculated the mAP, precision (P), recall (R), and F1 score using equations (1), (2), (3), and (4), respectively. Accordingly, 91.20% precision, 89.30% recall, and 90.24% F1 values were achieved. Moreover, the variations in P, R, and mAP performance criteria during the training process are illustrated in Figure 6. T_p, F_p, and F_n represent the terms true positive, false positive, and false negative, respectively.


$$P\;=\;\frac{T_{p}}{T_{p}+F_{p}}$$ (2)

$$R\;=\;\frac{T_{p}}{T_{p}+F_{n}}$$ (3)

$$F1=2*\left(\frac{P*R}{P+R}\right)$$ (4)

**Figure 7:**
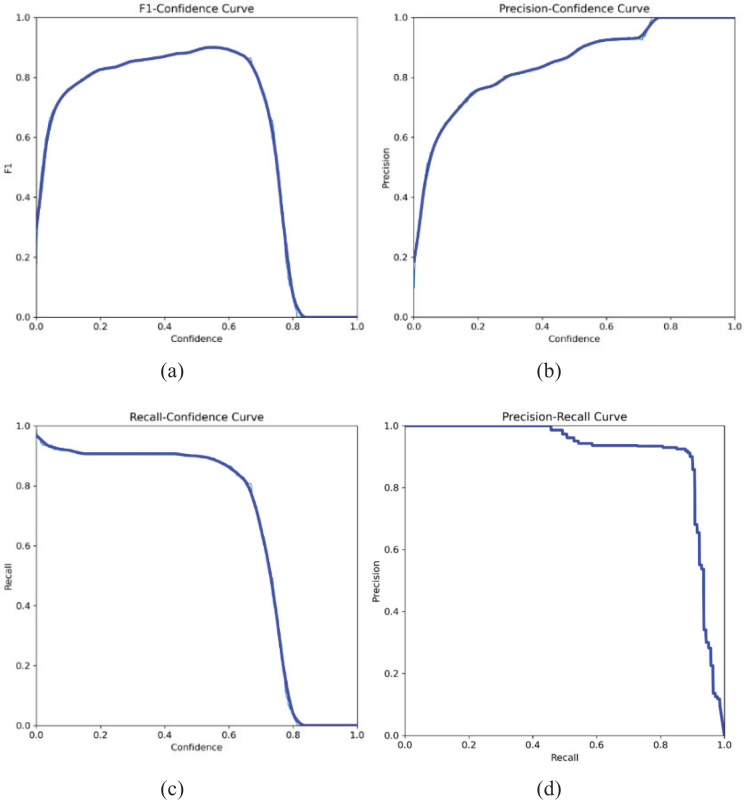
Determination of confidence interval based on (a) F1-score, (b) precision, (c) recall metrics, and (d) performance analysis of the detection model on the precision–recall curve.

The confidence value represents the detection probability and the accuracy of the bounding box as a percentage. The curves in Figure 7 were used to determine the most appropriate confidence interval of the presented model during the testing process.

It is reasonable to determine the confidence value in the range of 60% – 70% from the curves in Figure 7. Accordingly, the confidence value for the presented model in this study was determined as 70%. The area under the Precision-Recall curve in Figure 7d is proportional to the performance of the detection model. As the area increases, the performance of the model increases [19]. It can be seen from Figure 7d that the detection model offers satisfactory training performance.

### Test performance

The hyperparameters of the presented model are given in Table 2. The confusion matrix in Table 3 is provided for the test performance of the model trained on these parameters. While 135 of the 156 approximal caries labeled by the pediatric dentist were successfully detected with the proposed detection algorithm, 21 of them could not be detected. Furthermore, the YOLOV5-based detection algorithm was able to recognize five caries that was missed by the pediatric dentist during the labeling process. There are no approximal caries that is not labeled by the dentist and incorrectly detected by the developed model. P, R, and F1 metrics of the model were calculated from the confusion matrix in Table 3, respectively, *P* = 96%, *R* = 86%, and F1 = 91%.

**Table 2:** Hyperparameters.

Optimizer	Stochastic gradient descent (SGD)
Image size	640
Batch size	1
Execution environment	GPU
Learning rate	0.01
Momentum	0.937

GPU: Graphics Processing Unit

**Table 3:**
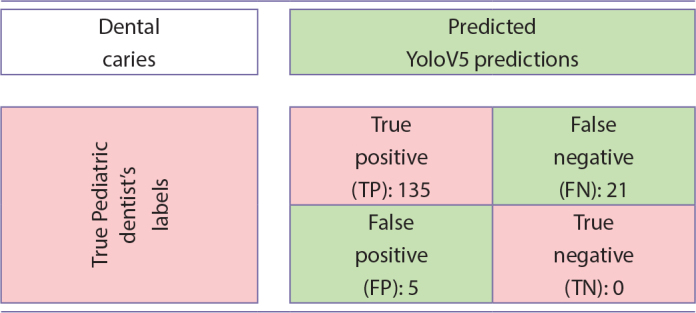
Confusion matrix from the test data.

## Discussion

Dental caries is one of the most common chronic diseases among children and adolescents. They significantly affect both oral and general health. In cases where dental caries is not diagnosed and treated, it progresses to the pulp tissue and causes pain [20]. It is also known that dental caries affects the quality of life related to oral and dental health in children [21]. For these reasons, early detection and diagnosis of approximal caries, which are difficult to detect visually, are important in children. Early diagnosed approximal caries can be stopped with preventive treatments.

Besides clinical examination, radiography plays an important role in the detection of dental caries, especially approximal and root caries [22]. In addition, numerous studies have reported large differences in the reliability and accuracy of detection, depending on the methodology of the study and the clinician’s level of experience [23]. Various methods are being developed and improved in the detection and diagnosis of dental caries to overcome the limitations of clinical and radiographic diagnosis [24]. However, unlike simple and traditional methods, deep CNN algorithms are systems that perform edge detection very efficiently. For this reason, the presented study aimed to determine approximal caries in pediatric patients with advanced detection algorithms based on CNN.

Kositbowornchai et al. [12] created artificial dental caries in a simulation environment and used artificial neural network-based Learning Vector Quantization (LVQ) to detect this caries. They also tried to estimate the depth of caries. They were more successful in detecting cavities than estimating the depth of caries. They detected caries with 81% accuracy on digital radiography images. We achieved 90.8% accuracy in caries detection with a CNN-based model. Using an advanced detection algorithm may have increased the accuracy of our model.

Lee et al. [24] tried to detect dental caries using deep CNN algorithms on periapical radiography images with a larger dataset. They preferred the GoogLeNet Inception v3 CNN network in their study and emphasized that the deep CNN structure is effective in detecting dental caries. They reached the 89% accuracy with GoogLeNet Inception v3 [24]. We used 13 different current YOLO builds for a similar problem. In our study, we compared a total of 13 detection algorithms, from YOLOv4 to the latest YOLOv8, for high performance, and in this way, we decided on the most suitable detection algorithm for the presented problem. At the end of the comparison, we reached an average of 90.8% accuracy with the YOLOv5-s. But with other YOLO builds we tried, we saw a maximum accuracy of 88.7%. Therefore, we can say that although the accuracy value we presented is higher than that of Lee et al., the accuracies for both studies are similar.

Chen et al. [25] performed tooth detection and numbering using an advanced CNN version. Using faster regions with convolutional neural network features (faster R-CNN) for rapid identification and numbering of teeth, they achieved 90% precision and 91% recall. We achieved 89.3% precision and 91.2% sensitivity. The use of up-to-date and advanced detection algorithms in both studies may have yielded similar results.

Geetha et al. [10] classified the presence of dental caries using the back-propagation neural network algorithm over 105 digital radiographic images. The system they presented gave 97% accuracy. When all of the studies are examined, it has been concluded that caries detection is done successfully with CNN-based algorithms. The use of CNN architecture has demonstrated considerable accuracy and efficiency in the diagnosis of suspected dental caries. However, despite these promising results, there are several limitations to the current study. To achieve a more accurate diagnosis of dental caries, clinical examinations that incorporate a comprehensive medical history, percussion, and tactile assessments, as well as radiographic examinations, should also be taken into consideration. The absence of clinical parameters in this study is considered a limitation, as it may not provide a complete picture of the patient’s dental condition. Artifacts on some radiographic images may have made it difficult for AI to detect caries. It is thought that higher accuracy and precision values will be achieved by increasing the dataset.

In this work, approximal caries of pediatric patients was effectively detected with an advanced CNN-based detection algorithm. A total of 13 models based on YOLOv4, YOLOv5, YOLOv6, YOLOv7, and the latest YOLOv8 have been trained on the original dataset created by collecting the radiographic images of pediatric patients aged 5–12 years. The trained models were tested with 20% of the dataset. The remaining data were used for training. The highest accuracy was achieved with the YOLOv5-based model as a result of the training. Performance metrics for this model were calculated as 91.2% precision, 90.8% mAP, 89.3% recall, and 90.24% F1 score, respectively. To the knowledge of the authors, approximal caries in primary teeth were tried to be detected for the first time in this study with all of the proposed CNN-based algorithms. It is thought that the detection model in this study will be supported by an approximal program to be used in various diagnostic processes in the future. Through the program planned to be developed, it can be ensured that the diagnosis time is reduced, the workload of the dentists is alleviated, faster and more effective diagnoses are made, and more patients can be treated at the same time. In addition, this study will provide infrastructure for future systems that are considered to be developed to detect various caries. The integration of AI-based detection models into clinical practice has significant potential to enhance diagnostic efficiency and accuracy. By integrating the AI-model into clinical workflows as a portable program, dentists can reduce the likelihood of overlooking cavities, leading to faster and more accurate diagnoses. However, barriers such as the cost of implementing such systems, the necessity for specialized training for clinical staff, and resistance due to unfamiliarity with AI technologies must be addressed to ensure seamless adoption.

The highlights of our work can be summarized as follows:

- In this work, the findings show that approximal caries, which is relatively difficult to diagnose in children, can be diagnosed with high accuracy through AI. According to our extensive literature review, we believe that this is the first study to successfully detect approximal caries on periapical films in primary teeth using AI.
- In pediatric dentistry, diagnostic time can be reduced by programs developed using the AI-based model presented in this paper.
- With the proposed detection model, the error rate of the pediatric dentist in diagnosis decreases. Since the detection model can reveal isolated dental caries, the number of caries that may be overlooked is minimized. The presented work is one of the strong examples in the engineering field of pediatric dentistry. Hence, the paper is also important as it is a multidisciplinary research.
